# Refining the electroclinical spectrum of *NPRL3*‐related epilepsy: A novel multiplex family and literature review

**DOI:** 10.1002/epi4.12798

**Published:** 2023-09-01

**Authors:** Alice Dainelli, Michele Iacomino, Sara Rossato, Samuela Bugin, Monica Traverso, Mariasavina Severino, Stefano Gustincich, Valeria Capra, Marco Di Duca, Federico Zara, Marcello Scala, Pasquale Striano

**Affiliations:** ^1^ Pediatric Neurology and Muscular Diseases Unit IRCCS Istituto Giannina Gaslini Genoa Italy; ^2^ Department of Neurosciences, Rehabilitation, Ophthalmology, Genetics, Maternal and Child Health Università Degli Studi di Genova Genoa Italy; ^3^ UOC Genetica Medica IRCCS Istituto Giannina Gaslini Genoa Italy; ^4^ U.O.C. Pediatria, Ospedale San Bortolo Vicenza Italy; ^5^ Neuroradiology Unit, IRCCS Istituto Giannina Gaslini Genoa Italy; ^6^ Italian Institute of Technology (IIT) Genoa Italy

**Keywords:** epilepsy, FCD, focal cortical dysplasia, focal seizures, frontal lobe, *NPRL3*

## Abstract

**Objective:**

*NPRL3*‐related epilepsy (NRE) is an emerging condition set within the wide GATOR‐1 spectrum with a particularly heterogeneous and elusive phenotypic expression. Here, we delineated the genotype–phenotype spectrum of NRE, reporting an illustrative familial case and reviewing pertinent literature.

**Methods:**

Through exome sequencing (ES), we investigated a 12‐year‐old girl with recurrent focal motor seizures during sleep, suggestive of sleep‐related hypermotor epilepsy (SHE), and a family history of epilepsy in siblings. Variant segregation analysis was performed by Sanger sequencing. All previously published NRE patients were thoroughly reviewed and their electroclinical features were analyzed and compared with the reported subjects.

**Results:**

In the proband, ES detected the novel *NPRL3* frameshift variant (NM_001077350.3): c.151_152del (p.Thr51Glyfs*5). This variant is predicted to cause a loss of function and segregated in one affected brother. The review of 76 patients from 18 publications revealed the predominance of focal‐onset seizures (67/74–90%), with mainly frontal and frontotemporal (32/67–47.7%), unspecified (19/67–28%), or temporal (9/67–13%) onset. Epileptic syndromes included familial focal epilepsy with variable foci (FFEVF) (29/74–39%) and SHE (11/74–14.9%). Fifteen patients out of 60 (25%) underwent epilepsy surgery, 11 of whom achieved complete seizure remission (11/15–73%). Focal cortical dysplasia (FCD) type 2A was the most frequent histopathological finding.

**Significance:**

We reported an illustrative *NPRL3*‐related epilepsy (NRE) family with incomplete penetrance. This condition consists of a heterogeneous spectrum of clinical and neuroradiological features. Focal‐onset motor seizures are predominant, and almost half of the cases fulfill the criteria for SHE or FFEVF. MRI‐negative cases are prevalent, but the association with malformations of cortical developments (MCDs) is significant, especially FCD type 2a. The beneficial impact of epilepsy surgery in patients with MCD‐related epilepsy further supports the inclusion of brain MRI in the workup of NRE patients.

**Key points**

- *NPRL3*‐related epilepsy (NRE) is a GATORopathy showing a peculiar phenotype signature, such as focal seizures with and without focal cortical dysplasia.
- The phenotype spectrum of NRE remains so far elusive, prompting a further detailed characterization to drive clinical management and therapeutical choices.
- We present a family with multiple affected subjects harboring a novel *NPRL3* truncating variant, including a patient with sleep‐related hypermotor epilepsy (SHE).
- A review of the literature unveiled that familial focal epilepsy with variable foci (FFEVF) and SHE are frequent epilepsy syndromes in NRE.
- Brain MRI in patients with NRE may help detect malformations of cortical development (MCDs), which may benefit from epilepsy surgery.

## 1 INTRODUCTION

*NPRL3* (nitrogen permease regulator 3‐like protein, OMIM * 600928) haploinsufficiency has recently emerged as a relevant cause of focal epilepsies.^1^ This gene encodes a 569‐amino acid protein, which is part of the GATOR1 complex (GAP Activity TOward Rags), together with DEPDC5 (DEP domain‐containing protein 5, OMIM * 614191) and NPRL2 (nitrogen permease regulator 2‐like protein, OMIM * 607072).^2^, ^3^, ^4^, ^5^ This complex is a negative regulator of mTORC1 (mammalian/mechanistic Target Of Rapamycin Complex 1), a kinase acting as a critical regulator of protein synthesis, transcription, cell growth, metabolism, and death.^6^, ^7^, ^8^ The mTORC1 kinase also plays pivotal brain‐specific roles, especially in the regulation of synaptic plasticity and neurogenesis.^9^

In the past decade, the involvement of mTOR hyperactivation in the pathogenesis of several conditions featuring brain malformations and epilepsy (eg, tuberous sclerosis, hemimegalencephaly, and focal cortical dysplasia [FCD]) has been widely investigated. The pathogenic relevance of variants affecting the function of mTOR inhibitors such as GATOR1 has subsequently emerged.^10^, ^11^, ^12^ Supporting examples include loss‐of‐function variants in *NPRL3*, *DEPDC5*, and *NPRL2*.^13^, ^14^, ^15^, ^16^ Noteworthy, similar variants have also been associated with focal epilepsy without frank brain abnormalities,^14^, ^17^ and the genes encoding for members of the GATOR1 complex are the most frequently mutated in focal epilepsies.^18^

Although a growing interest has recently emerged about the role of mTORopathies in epilepsy, only 35 *NPRL3* pathogenic and likely pathogenic variants have been reported in the literature so far. In this study, we report a novel *NPRL3* variant segregating within a family with multiple affected individuals with epilepsy and reviewed the pertinent literature about *NPRL3*‐related epilepsy (NRE), providing a detailed overview of the genotype and phenotype spectrum of this condition.

## 2 METHODS

### 2.1 Clinical case study

The study was approved by the local ethics committee and conducted following the Helsinki Declaration. A retrospective review of the clinical charts and EEG recordings was performed, and details were collected about developmental and past medical history, epilepsy history, pharmacological treatments, and results from other relevant diagnostic investigations. Informed consent was obtained from the patient's parents for genetic testing and data publishing.

### 2.2 Genetic studies

Exome sequencing (ES) was performed on genomic DNA extracted from peripheral blood of the proband and the parents. 37 Mb of genomic DNA (gDNA) including exons and splicing sites of around 19 000 genes were enriched using the kit Nextera Rapid Capture Exome and analyzed through massively parallel sequencing (Illumina, PE 2x150). The mapping of the sequences was done using GATK software. The selection of most plausible candidate variants was made according to allelic frequency (<0.001) in population dataset (gnomAD), presence in ClinVar, conservation of the affected residues (GERP), and predicted impact on protein structure and function by in silico tools (CADD, Mutation Taster, PolyPhen‐2, SIFT).^19^ Candidate variants were eventually classified according to the ACMG‐AMP criteria (Richards et al.),^20^ and Sanger sequencing was performed for their validation and segregation in family members.

### 2.3 Extraction of NPRL3 variants and case descriptions from the literature

We systematically reviewed the studies on NRE published in PubMed (https://pubmed.ncbi.nlm.nih.gov/pubmed; accessed January 2023), using the following terms: “*NPRL3* AND seizures” (28 results) and “*NPRL3* AND epilepsy” (44 results). The abstracts of the retrieved references were reviewed and prioritized based on the relevance of the content and the quality of the reported evidence. Furthermore, we used the reference lists of the selected articles to search for additional pertinent papers. We included only articles in English and studies conducted on humans and excluded those studies in which *NPRL3* variants were not tested or were found not to be associated with the phenotype. Studies not reporting single‐patient clinical data were excluded as well. Demographic and genetic data, seizure history, EEG, and neuroimaging reports, when available, were extrapolated and thoroughly reviewed. After this selection, 18 studies were used for data collection and review.

## 3 RESULTS

### 3.1 Case description

#### 3.1.1 II‐5

**FIGURE 1:**
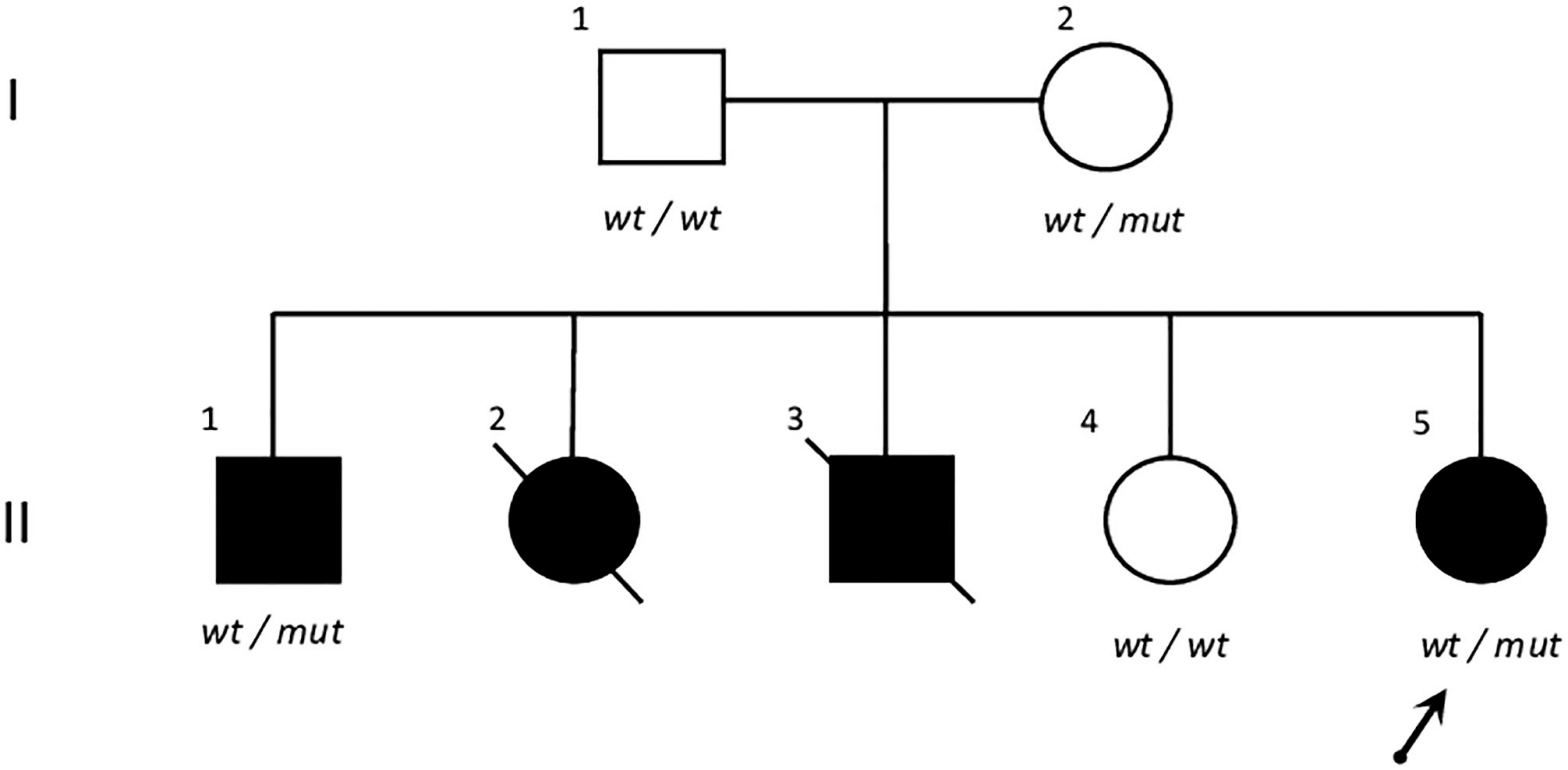
Pedigree of the reported family. The proband is indicated by an arrow. Affected individuals and asymptomatic subjects are indicated by shaded or empty symbols, respectively. Where available, the segregation of the wild‐type (wt/wt) and mutant (wt/mut) *NPRL3* allele is indicated.

The proband is a 12‐year‐old girl (II‐5) (Figure 1) born at 38 weeks of gestation to unrelated healthy parents from Senegal. The pregnancy and neonatal course were uneventful. The patient regularly met the developmental milestones in the first year of life. At the age of 11 months, she had a complex febrile seizure in the context of respiratory syncytial virus (RSV) pneumonia. The following day, she experienced two additional episodes of fever‐induced, self‐limiting generalized seizures, lasting around 5 minutes. At the age of 4, the patient developed persistent daily focal motor seizures (Table 1).

**TABLE 1:** Electroclinical features of the reported *NPRL3* patients.

Patient ID	Febrile seizures	Epilepsy onset (years)	EEG	Brain MRI	Daytime seizures	Nocturnal seizures
II‐5	Yes	4	Left frontal	Normal	Focal motor seizures with deviation of head and mouth to the right, associated with vocalization. Longer episodes (20–30 s) with impaired awareness and sphincter release also occurred	Characterized by a scream awakening the patient, followed by tonic eye deviation and deviation of head and mouth to the right (lasting about 10 s)
II‐1	No	15	Frontal bilateral	Normal	Loss of awareness, clonus of the four limbs, and flexion of the trunk, without fall	Generalized jerks, sialorrhea, *morsus*, tonic eye deviation to the right
II‐2	Yes	5	Left frontotemporal	Normal	Sudden loss of awareness	Generalized tonic–clonic seizures
II‐3	Yes	6	Left frontotemporal	Normal	Loss of awareness, eyelid clonus, staring, head twist to the right	Prolonged generalized tonic–clonic seizures

Abbreviations: EEG, electroencephalogram; MRI, magnetic resonance imaging.

**FIGURE 2:**
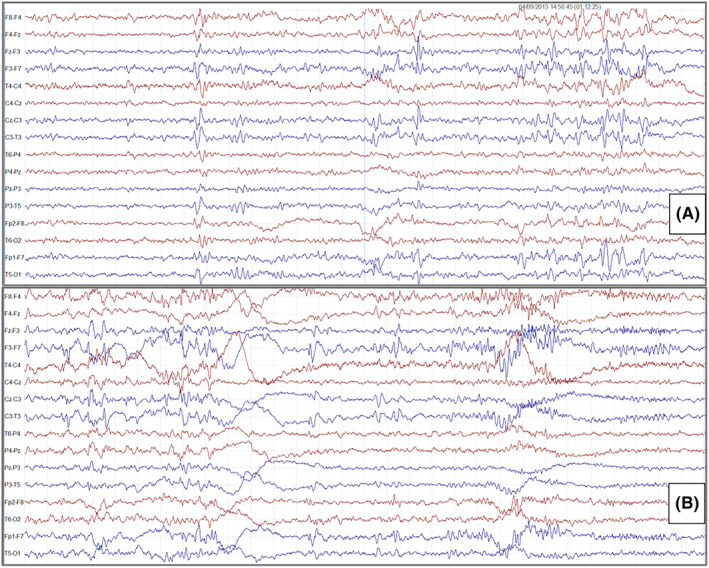
EEG recordings of the proband (II‐5). Amplitude 10 μV/mm; TC:0.10 s‐HF 30.0 Hz. A, EEG during nREM sleep (phase 1) shows increased paroxysmal activity with high‐voltage spikes/slow‐wave complexes over the anterior regions of both hemispheres with a relatively well‐organized sleep structure. B, EEG during nREM sleep (phase 2) shows paroxysmal activity with high‐voltage spikes/slow‐wave complexes over the anterior and central areas in both hemispheres and low‐voltage fast rhythms on the right frontal and central areas.

**FIGURE 3:**
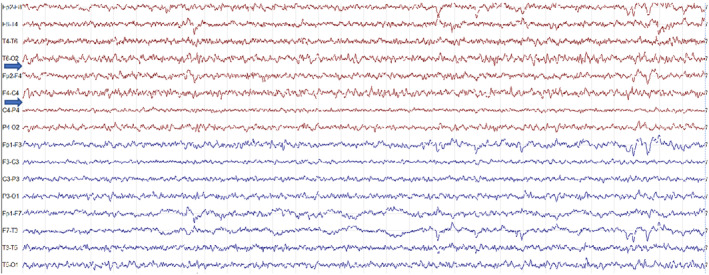
EEG recordings of the proband (II‐5). Amplitude 10 μV/mm; TC:0.10 s‐HF 30.0 Hz. Rest EEG at the age of 4 showing mid‐amplitude polymorphic delta activity intermixed with wave/slow‐wave complexes over the right hemisphere (arrows).

**FIGURE 4:**
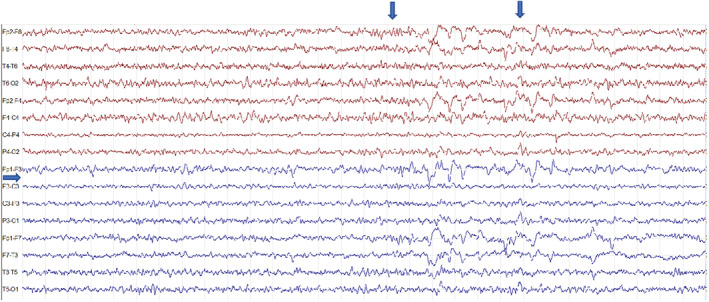
EEG recordings of the proband (II‐5). Amplitude 10 μV/mm; TC:0.10 s‐HF 30.0 Hz. During drowsiness, the EEG shows increased slowed rhythms and high‐voltage wave/slow‐wave complexes over the central and anterior right regions with diffusion on the left anterior brain regions (arrows).

These episodes lasted a few seconds and were characterized by deviation of head and mouth to the right, associated with vocalization. Longer episodes lasting up to 20–30 seconds characterized by loss of awareness and sphincter release, were observed. The patient also experienced nocturnal seizures lasting about 10 seconds. These latter episodes started with a scream, which awakened the patient, and were characterized by tonic eye deviation and deviation of head and mouth to the right. Prolonged video‐EEG monitoring showed a normal pattern during wakefulness, while interictal epileptiform discharges were noticed in the left anterior regions during sleep (Figures 2, 3, 4).

Ictal EEG showed broad sharp waves in the frontal regions occurring seconds before the clinical seizure, followed by the spreading of paroxysmal activity to the whole left hemisphere. Brain magnetic resonance imaging (MRI) was normal. Carbamazepine (20 mg/kg/day) was effective in controlling seizures. Three months after carbamazepine was started, sleep‐EEG showed bilateral epileptic activity in the frontal regions, lasting less than a minute and consisting of flexion‐abduction movements of lower limbs and upper limbs automatisms, which resolved spontaneously. Carbamazepine dosage was thus increased (30 mg/kg/day), and subsequent EEGs did not show any abnormal electrical activity.

#### 3.1.2 II‐1

This subject did not suffer from febrile seizures and had normal psychomotor development. At the age of 15 years, he developed seizures characterized by impaired awareness, jerking of the four limbs, and flexion of the trunk. The patient was treated with carbamazepine (20 mg/kg/day), leading to good seizure control. However, treatment was soon stopped due to patient's decision and sleep‐related convulsive seizures relapsed. EEG showed bilateral anterior abnormalities during nREM sleep. Brain MRI was normal.

#### 3.1.3 II‐2

This patient presented with febrile seizures at 4 years, during a pneumonia episode. When she was 5 years old, she experienced brief episodes of sudden loss of awareness. Brain MRI was normal. EEG showed interictal epileptiform discharges in the left frontotemporal regions, and valproic acid treatment (15 mg/kg/day) was started. Nevertheless, the girl experienced nocturnal hyperkinetic episodes, suggestive of frontal nocturnal epilepsy (or sleep‐related hypermotor epilepsy (SHE), and carbamazepine (20 mg/kg/day) treatment was therefore started. When treatment was subsequently modified for pregnancy planning, seizures increased in frequency and changed in semiology, switching to sudden awakening, screaming, and apnea or hyperventilation. The patient died at the age of 27, after a prolonged nocturnal seizure.

#### 3.1.4 II‐3

This patient had normal psychomotor development. He suffered from simple febrile seizures at the age of 2 years. From the age of 6 years, seizures occurred both during daytime and nighttime. Diurnal seizures consisted of loss of awareness, staring, eyelid myoclonia, and right head version. Sleep‐related episodes mainly occurred as long bilateral tonic–clonic seizures. EEG showed left frontotemporal anomalies. Seizures were refractory to carbamazepine (30 mg/kg/day). He died at the age of 11 years, during a prolonged convulsive status epilepticus.

### 3.2 Genetic findings

Exome sequencing led to the identification of the heterozygous variant NM_001077350.3: c.151_152delGT (p.Thr51Glyfs*5) in *NPRL3* in II‐5. Segregation analysis revealed that the variant is present in her older brother (II‐2) and mother. This variant causes the deletion of the Thr51, leading to a frameshift which likely results in nonsense‐mediated mRNA decay (NMD) or the formation of a truncated transcript. Hitherto, the (p.Thr51Glyfs*5) is predicted to cause a loss of protein function. The variant is absent in gnomAD and ClinVar and is predicted pathogenic according to ACMG/AMP guidelines (criteria PVS1, PM2, and PP3). The identification of the variant in two affected siblings (II‐5 and II‐2) suggests an incomplete penetrance in the context of an autosomal dominant inheritance pattern. No other potentially damaging variants in known disease‐related genes were detected.

### 3.3 Literature review

Eighteen publications were selected. A total of 76 patients with a history of seizures and harboring *NPRL3* variations were reviewed. The reported subjects carried pathogenic or likely pathogenic single nucleotide variants and micro‐deletions. Comprehensively, 35 different *NPRL3* variations were identified (Table 2). Unaffected subjects harboring *NPRL3* variants were not considered since a non‐systematic data extraction was performed and limited information about these individuals was available in the literature. In the widest pedigree of NRE (n = 133) reported so far,^21^ 36.1% of subjects had a history of seizures, with an estimated penetrance of 28% based on 10 different families (with sibships comprehensively tested for *NPRL3* mutation) out of the whole cohort. In other *NPRL3*‐mutated families, seizure penetrance ranged from 33% to 50%^14^, ^15^, ^17^, ^22^ (Table 2).

**TABLE 2:** Genetic and clinical features of subjects with *NPRL3*‐related epilepsy.

Chronological order of publication of novel variants/gene deletions	*NPRL3* cDNA variant (NM_001243247.1)	Protein alteration	Epilepsy—seizure type	EEG	MRI —histopathological findings	Reference
1.	c.835_836insT	p.(Ser279Phefs*52)	SHE	N/A	N/A	Ricos et al.^15^
			SHE			
			Neonatal seizures			
			Febrile seizures			
2.	c.1376_1377insAC	p.(Ser460Profs*20)	TLE	N/A	N/A	Ricos et al.^15^
			TLE			
			Nocturnal tonic–clonic seizures			
3.	c.745G > A	p.(Glu249Lys)	FE	N/A	N/A	Ricos et al.^15^
4.	c.275 G > A	p.(Arg92Gln)	FLE	N/A	N/A	Ricos et al.^15^
5.	c.954_955insCCCA	p.(Trp319Profs*13)	TLE	N/A	N/A	Ricos et al.^15^
6.	c.1375_1376dupAC	p.(Ser460Profs*20)	Unclassified	Suppression burst	FCD IIa	Sim et al.^14^
			FE	Left frontal sharp waves	Normal	Sim et al.^14^
			FE	Ictal rhythms and postictal slowing over the right frontal region	Bottom‐of‐sulcus dysplasia (FCD IIa)	Sim et al.^14^
			FE	Focal interictal epileptiform discharges, and seizures of right frontal origin	Normal	Sim et al.^14^
7.	c.1352‐4 delACAGinsTGACCCATCC	—	FE	Left central interictal and ictal abnormalities	FCD IIa	Sim et al.^14^
Recurrent variant	c.275G > A	p.(Arg92Gln)	FE with tonic–clonic seizures	Continuous focal epileptiform discharges and ictal rhythms from the left centroparietal region	FCD IIa	Sim et al.^14^
8.	c.1522delG	p.(Glu508Argfs*4)	SHE	Right temporal sharp waves (4 y), right frontal sharp slow waves (7 y) frontocentral spikes (20 y)	Normal	Korenke et al.^22^
			SHE	Precentral, temporal sharp waves (4 y) Precentral sharp waves (12 y)	Normal	Korenke et al.^22^
			SHE	N/A	Normal	Korenke et al.^22^
9.	c.1270C > T	p.(Arg424*)	Febrile seizures epilepsy with tonic–clonic seizures	Diffuse irregular SWC with changing maximum	N/A	Weckhuysen et al.^16^
			Epilepsy with tonic–clonic seizures	Diffuse irregular SWC with changing maximum	Normal	Weckhuysen et al.^16^
			FFEVF	Sharp waves, predominantly left temporal	N/A	Weckhuysen et al.^16^
			FFEVF	Interictal EEG was normal Ictal EEG was not localizing	Normal	Weckhuysen et al.^16^
			FFEVF	Frontocentral left spikes. SEEG (5y): left mesial temporal onset	FCD IIa and hippocampal sclerosis	Weckhuysen et al.^16^
10.	c.1070delC	p.(Pro357Hisfs*56)	FFEVF	N/A	N/A	Weckhuysen et al.^16^
	c.1070delC	p.(Pro357Hisfs*56)	FFEVF	Right frontocentral discharges and ictal activity in the right centroparietal area.	normal	Weckhuysen et al.^16^
	c.1070delC	p.(Pro357Hisfs*56)	Unclassified	Normal	N/A	Weckhuysen et al.^16^
	c.1070delC	p.(Pro357Hisfs*56)	FFEVF	Interictal EEG showed bilateral spikes in the frontal and vertex area. Ictal EEG showed fast ictal activity with onset in the left frontal lobe.	FCD IIB	Weckhuysen et al.^16^
11.	c.301C > T	p.(Gln101*)	FE	Epileptiform abnormalities over the left centro‐parietal region	Normal	Baldassari et al.^18^
12.	c.493delC	p.(Arg165Glyfs*5)	FE	N/A	Normal	Baldassari et al.^18^
13.	c.562C > T	p.(Gln188*)	SHE	Right frontal epileptic discharges	Normal	Baldassari et al.^18^
Recurrent variant	c.1270C > T	p.(Arg424*)	SHE	Right frontal epileptic discharges	Normal	Baldassari et al.^18^
14.	c.1557C > G	p.(Tyr519*)	SHE	Left frontal epileptic discharges	Normal	Baldassari et al.^18^
15.	Deletion (exons 5‐10)	p.(?)	FLE	Interictal: initial EEGs with right and later EEGs with left frontocentral spikes. Ictal: left frontocentral paroxysmal activity during both sleep and awakening	Normal	Baldassari et al.^18^
16.	Deletion (exons 1‐7)	p.(?)	FLE	Interictal: left central epileptiform abnormalities. Ictal: left central discharges.	Normal	Baldassari et al.^18^
17.	Partial 38‐kb deletion comprising eight exons (exons 8‐15) and the 3′UTR of the *NPRL3*	—	FE (FFEVF)	No IED, intermittent bilateral posterior and right frontal slowing	Normal	Canavati et al.^33^
		—	FE (FFEVF)	N/A	N/A	Canavati et al.^33^
		—	SHE (FFEVF)	N/A	N/A	Canavati et al.^33^
		—	FLE (FFEVF)	Right frontal IED, intermittent bilateral parietal slowing	N/A	Canavati et al.^33^
		—	FE (FFEVF)	N/A	N/A	Canavati et al.^33^
		—	Unclassified (FFEVF)	No IED, intermittent left frontotemporal slowing	N/A	Canavati et al.^33^
		—	Unclassified (FFEVF)	No IED, intermittent left posterior, and generalized slowing	N/A	Canavati et al.^33^
		—	Unclassified (FFEVF)	Normal	Normal	Canavati et al.^33^
		—	FE (FFEVF)	N/A	N/A	Canavati et al.^33^
		—	FE (FFEVF)	IED, generalized and right hemisphere slowing	HME	Canavati et al.^33^
18.	c.1063C > T	p.(Gln355*)	Unclassified (FFEVF)	Left frontotemporal slowing	N/A	Canavati et al.^33^
			FE (FFEVF)	Fast activity in the left frontotemporal and central region	N/A	Canavati et al.^33^
			FE (FFEVF)	Diffuse slowing more prominent on the left	N/A	Canavati et al.^33^
			FE (FFEVF)	Diffuse slowing	N/A	Canavati et al.^33^
			Unclassified Bilateral convulsive seizures (FFEVF)	N/A	N/A	Canavati et al.^33^
19.	Whole gene deletion 16p13.3(93 722_646 006)	—	Infantile spasms; focal motor seizures	Burst suppression pattern; spikes and sharp waves over left central regions	Unclassified large left hemisphere cortical malformation	Vawter‐Lee et al.^26^
20.	c.349delG	p.(Glu117Lysfs*)	48‐patients pedigree No single‐patient data available	Both focal (spikes, sharp waves) and generalized (generalized spike‐and‐wave discharges, and/or slowing) abnormalities	8 FCD, 1 HME	Iffland et al.^21^ Iffland et al.^32^
21.	c.1504C > G	p.(Pro502Ala)	Unclassified	N/A	N/A	Dunn et al.^35^
22.	c.905C > T	p.(Pro302Leu)	FLE	Ictal: bilateral frontal Interictal: no changes	Normal	Krenn et al.^28^
23.	380‐kb microdeletion involving NPRL3 16p13.3(97 430_476 719)	—	Unclassified focal epilepsy	Interictal: right frontotemporal spikes	Normal	Krenn et al.^28^
24.	c.898C > T	p.(Gln300*)	FLE	Ictal: not localizable Interictal: no changes	Normal	Krenn et al.^28^
25.	c.1561G > A	p.(Ala521Thr)	TLE	N/A	Normal	Krenn et al.^28^
Recurrent variant	c.745G > A	p.(Glu249Lys)	TLE	N/A	Normal	Krenn et al.^28^
26.	c.1053G > C	p.(Gln351His)	FE	N/A	Normal	Krenn et al.^28^
27.	—	p.(Ser460Profs*20)	Unclassified	N/A	FCD IIA	Lee et al.^41^
28.	c.898_900del	—	FE	Ictal onset in the left central area	FCD IIA	Benova et al.^50^
			FE	N/A	Normal	Benova et al.^50^
Recurrent variant	c.1270C > T	p.(Arg424*)	Unclassified	Frontal and temporal epileptic foci	Normal	Abumurad et al.^27^
29.	c.1149dupC	p.(Ala384fs)	N/A	N/A	Polymicrogyria	Blümcke et al.^29^
30.	3‐kb deletion involving *NPRL3* 16p13.3(161 898_164 745)	—	Unclassified	Consistent with early infantile epileptic encephalopathy	HME	Chandrasekar et al.^23^
31.	c.316C > T	p.(Gln106*)	FFEVF FBTCS	Normal	Brain atrophy	Li et al.^17^
			FFEVF TLE FBTCS	Sharp and slow‐wave complex predominantly in right temporal lobe	Normal	
			FFEVF FBTCS	Normal	Normal	
			FFEVF FBTCS	Normal	Normal	
			FFEVF FLE FBTCS	Sharp waves in the right frontal lobe	Normal	
32.	c.48delG	p.Ser17Alafs*70	FE	Spike‐and‐wave activity in the right frontocentral region, spread to bilateral frontocentral regions with low‐amplitude fast activity evolving to rhythmic theta activity	FCD II a	Bennett et al.^30^
			Epileptic spasms FE	Left frontal epileptiform discharges	FCD II a	
33.	c.954C > A	p.Y318*	FFEVF	Diffuse irregular spike‐waves in the left anterior hemisphere	Normal	Hu et al.^36^
			FFEVF	Spike‐slow‐wave complexes in the left posterior region		
34.	c.1545‐1G > C	Splicing variant	FFEVF	Spike‐slow‐wave complexes in left central, parietal, and midline areas	Cortical thickening of the left frontal gyrus	
35.	c.151_152del	(p.Thr51Glyfs*5)	SHE	Left frontotemporal epileptiform discharges	Normal	Our study
			SHE			

**FIGURE 5:**
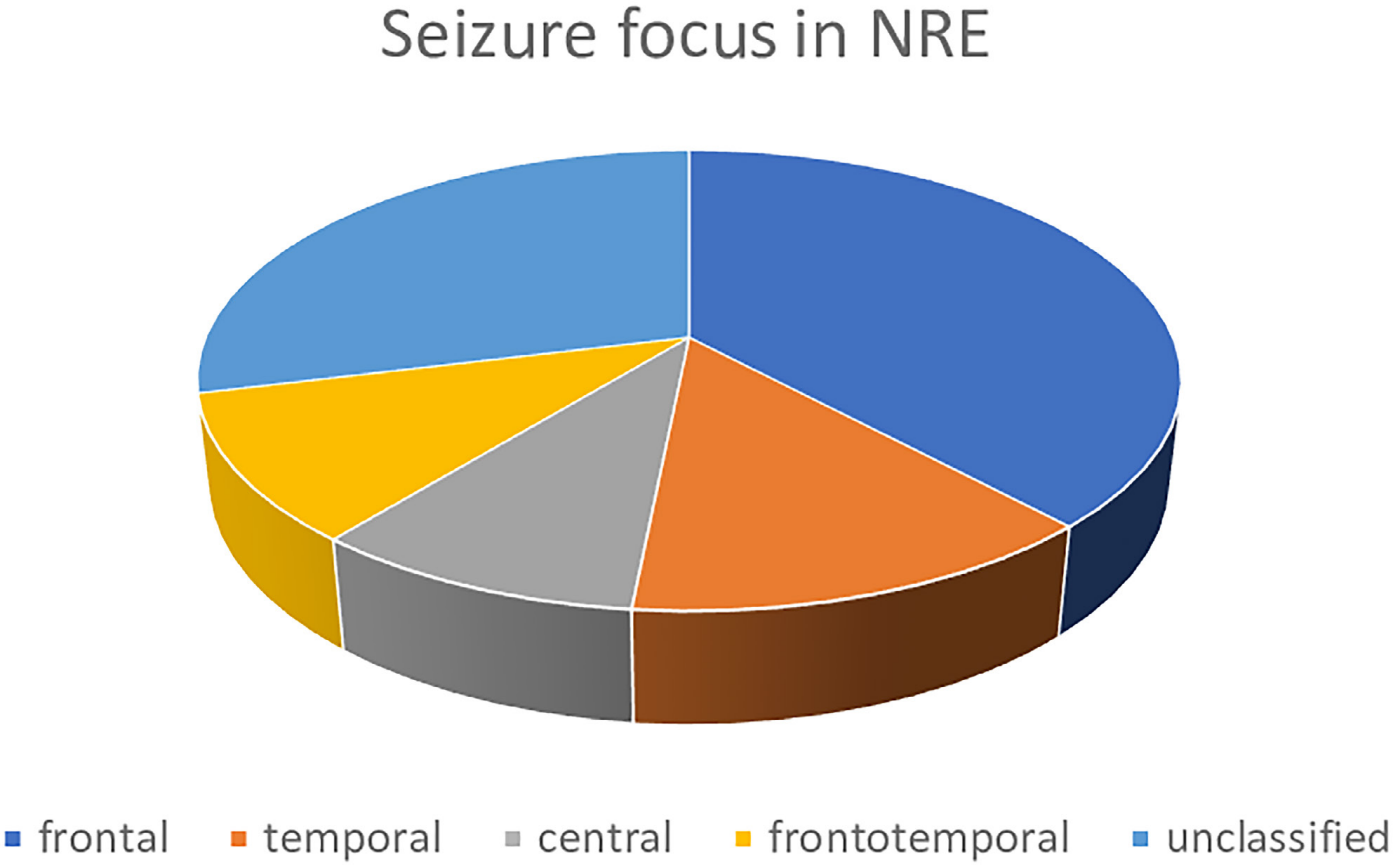
Schematic spectrum of seizure focus in patients with *NPRL3*‐related epilepsy (NRE). Frontal‐onset seizures are predominant, followed by temporal, central, and frontotemporal. In a large proportion of patients seizure focus remains unclear.

Clinical reports either describing seizure semiology or reporting the epilepsy diagnosis and/or EEG findings were available for 74 patients. Affected females are predominant (63.8%) among *NPRL3* epileptic patients. The mean age at onset is 8.5 years (SD ± 9.7, median 6 years), with a range of 1 day to 51 years. Developmental delay was observed in five patients, language delay in three, and mild cognitive impairment in two adult patients (one aged 41 with a normal 3 T MRI and multiple weekly seizures, and the other aged 74 with age‐related brain atrophy and rare seizures).^17^ Combining clinical and EEG findings, focal‐onset seizures were the most common clinical manifestations (67/74–90%). Among these, frontal lobe onset seizures were predominant (25/67–37%), followed by “unspecified onset” focal seizures, either with no clear localization of seizure onset or with no ictal EEG findings (19/67–28%) (Figure 5; Table 3). Less common onsets were temporal (9/67–13%), frontotemporal (7/67–10%), and central (6/67–9%). Both seizures with secondary generalization (10/74–13.5%) and generalized seizures without any apparent focal onset in ictal EEG (9/74–12%) were reported.

**TABLE 3:** Clinical features and outcomes of *NPRL3* patients.

EEG focus	% (n)
Frontal	37 (25/67)^a^
Temporal	13 (9/67)
Central	9 (6/67)
Frontotemporal	10 (7/67)
Unclassified	28 (19/67)
EEG lateralization in focal‐onset seizures	% (n)
Monolateral	83 (34/41)^b^
Bilateral	17 (7/41)
Epilepsy syndromes	
SHE	14.9 (11/74)^c^
FFEVF	39 (29/74)
Histopathology	
FCD2a	60 (9/15)^d^
HME	6.6 (1/15)
MRI	
FCD	20 (10/49)^e^
HME	4 (2/49)
Polymicrogyria	2 (1/49)
Unclassified	6 (3/49)
Normal	67 (33/49)
AEM response	
Seizure‐free on 1 ASMs	18 (11/60)^f^
Seizure‐free on 2 ASMs	5 (3/60)
Global seizure freedom on ASMs	28 (17/60)
Surgery	
Yes	25 (15/60)
Complete seizure remission	66.6 (10/15)

^a^ 67: NRE patients with focal‐onset seizures.

^b^ 41: NRE patients with focal‐onset seizures and EEG findings reporting whether ictal onset rhythms are mono−/bilateral.

^c^ 74: NRE patients with available clinical and/or EEG data.

^d^ 15: NRE patients who underwent epilepsy surgery.

^e^ 49: NRE patients with available MRI data.

^f^ 60: NRE patients with available data on treatment.

EEG recordings in NRE patients have been detailed in 46 cases (49/76–64%). EEG findings included focal epileptiform discharges in frontal lobes, central areas, frontocentral areas, and temporal lobes. These abnormalities included sharp waves, spikes, and spike–wave complexes. Bilateral or generalized abnormalities have been reported as well. The reported patient with hemimegalencephaly^23^ showed an EEG with excessive discontinuity and burst suppression with inter‐burst intervals lasting less than 5 seconds.

Only two types of epilepsy syndromes have been described in patients with NRE: sleep‐related hyperkinetic epilepsy (SHE) and familial focal epilepsy with variable foci (FFEVF). Nocturnal frontal lobe epilepsy (NFLE) is here referred to as SHE, according to the updated definition proposed by Tinuper^24^ and later in the position paper on Classification and Nomenclature of Epilepsy Syndromes with onset at variable ages.^25^ SHE was diagnosed in 11/74 (14.9%) subjects with *NPRL3* causative variants as well as in two affected siblings without genetic testing for *NPRL3*. Familial focal epilepsy with variable foci was reported in three multiplex families with cases of temporal lobe, frontotemporal, and frontal lobe epilepsy.^16^, ^17^ These cases account for 39.2% (29/74) of the phenotypes in the *NPRL3* cohort. Among all reported patients, probable SUDEP was only reported in one patient.^16^

Among the 62 patients with available treatment information, 15 patients with focal lesions detected in MRI (except one with a normal MRI) and drug‐resistant epilepsy underwent epilepsy surgery. Lesionectomy was performed in nine subjects (14.5%), with an MRI suspect of FCD and a subsequent histopathological finding of FCD type 2a. Additional interventions included functional hemispherectomies in a hemimegalencephaly case and a case with a broad left hemisphere malformation (2/62–3%),^23^, ^26^ laser interstitial thermal therapy (LITT) on frontal and temporal foci (1/62–1.6%),^27^ deep brain stimulation (DBS) in a case of drug‐resistant epilepsy with no localizable EEG finding and normal MRI (1/62, 1.6%),^28^ and epileptic focus resection in a patient with polymicrogyria (1/62–1.6%).^29^ The outcomes of FCD resection were good, with six out of nine patients being seizure‐free off therapy and one seizure‐free on monotherapy (oxcarbazepine). One patient from this group still experiences sporadic seizures postoperatively while on treatment with oxcarbazepine, likely due to residual dysplastic tissue.^30^ Seizures also completely resolved in patients treated with hemispherectomy and LITT.^27^ Conversely, seizure freedom has been achieved neither in the case of a DBS implant^28^ nor in a polymicrogyria resection,^29^ likely possibly due to a wide extension of the primitive lesion and partial exertion. A recent paper confirmed that epilepsy surgery may be effective in some children with *GATOR1* complex gene variants, including *NPRL3* and seizure outcomes may be compromised by extensive epileptogenic zones.^31^

Histopathological findings in NRE are heterogeneous. FCD type 2A was observed in 60% (9/15) of patients undergoing epilepsy surgery, whereas FCD type 2B was suspected in a single case based on suggestive MRI features (ie, abnormal gyration, increased cortical thickness, and linear hyperintensity at the gray‐white matter junction in the right frontoparietal area).^16^ Globally, FCDs are the most frequent malformations of cortical development (MCDs) associated with NRE, especially in light of the eight additional recently reported FCD cases.^32^ Indeed, FCDs constitute around 60% of the *NPRL3*‐associated MCDs, being reported in 10/15 subjects with proven or suspected MCD on the brain MRI. Less common structural abnormalities include hemimegalencephaly,^23^, ^32^, ^33^ polymicrogyria,^29^, ^32^ and hippocampal sclerosis.^16^

Information regarding antiseizure medications (ASMs) was available for 60/76 patients. In the whole cohort, monotherapy was the most common regimen (21/60 patients—35%). Employed ASMs included carbamazepine in 11 patients, oxcarbazepine in 5, valproic acid in 3, lamotrigine in 2, phenobarbitone in 2, topiramate in 2, and levetiracetam in 1 (combined with ketogenic diet). Eight out of these 21 subjects (38%) were seizure‐free. Two ASMs were employed on eight patients, three of whom were seizure‐free. In one of these subjects, seizures were only remitted after epilepsy surgery. Three or more ASMs were administered in 19 patients and sirolimus was also prescribed in two subjects. None of these individuals achieved seizure freedom with medications but improved after surgery. Globally, seizure freedom on ASMs was achieved in 28% of cases (17/60) (Figures 4 and 5).

## 4 DISCUSSION

We report a novel family with multiple affected individuals presenting with NRE. The epileptic manifestations of these subjects match the phenotype traced in previously described cases, consisting of childhood‐onset and preferentially focal motor seizures originating in the frontal regions. Additionally, these seizures were more common during sleep and occasionally showed secondary generalization. No MCDs could be observed in our cases. However, brain abnormalities may be very subtle in *NPRL3* patients^16^ and could benefit from high‐field‐strength MR imaging.^34^

One of the major difficulties with *NPRL3* variants, as with other MTOR pathway‐dominant genes, is that the incomplete penetrance and variable expressivity make it difficult to establish causality. Even more so if we consider that not only is phenotypic variability observed among different variants, but also within the same variant (see c.275 G > A, c.1270C > T, Table 2). It is not possible to dissect how much of the phenotype is directly caused by the *NPRL3* variant. In our case, we did not detect any additional variants of possible interest in known disease genes, thus supporting the role of the *NPRL3* variant in determining the observed phenotype. The genetic background of the subject may certainly influence the expression of a specific variant and further studies will play a crucial role to investigate this aspect further.

Wider genomic testing (whole‐exome sequencing and whole genome sequencing) might prove helpful to rule out other gene candidates, which may be contributing to *NPRL3* pathogenic mechanism or rather constitute alternative more plausible causes. To this regard in NRE, exome sequencing has been applied in 10 studies (10/18–56%, including our case), with no additional pathogenic or likely pathogenic variants reported,^14^, ^15^, ^17^, ^22^, ^28^, ^30^, ^35^, ^36^ except for one case with epilepsy, hemimegalencephaly and multiple cavernomas where the additional PDCD10 gene variant suggested a digenic contribution to the heterogeneous phenotype.^33^ Differently, whole‐genome sequencing has only been reported once in the *NRE* cohort,^23^ where it allowed us to identify a causative deletion in *NPRL3*, but no other CNVs or gene variants were observed. Despite the so far timid results in identifying multiple variants and oligogenic mechanisms, a broader resort to whole‐genome sequencing together with deep phenotyping will help unveil the variability of presentations in NRE.

The *NPRL3* variants so far reported in patients (total = 29, excluding gene deletions) are mainly frameshift variants (23/29; 79%, Table 2), implying a loss of function effect (LoF) on the gene product. As already observed by Baldassari,^18^ missense variants in the GATOR1‐complex genes are the most frequent in gnomAD, while the population of patients with epilepsy shows enrichment in LoF. Nonetheless, missense variants have been reported as well in NRE (6 variants; 6/29; 21%), classified as variants of uncertain significance (*VUS*s) (p.Glu249Lys, p.Arg92Gln, p.(Ala521Thr), p.(Gln351His)) and likely pathogenic (p.(Pro302Leu)), according to Baldassari's algorithm for GATOR1 variants classification. These missense variants are associated with a heterogeneous phenotypic spectrum which does not differ from that related to truncating variants: ranging from drug‐responsive focal epilepsy alone, through frontal lobe epilepsy with intellectual disability, to drug‐resistant focal epilepsy with FCD and language delay. Indeed, in a pioneering and thorough revision of GATOR1 variants (reported until 2018), Baldassari et al. could not identify a clear‐cut genotype (LoF vs *VUS* or likely pathogenic variants)—phenotype (drug‐responsive focal epilepsy vs drug‐resistant epilepsy + MCD) correlation. Functional studies investigating the effect of such missense variants, quantifying mTORC1 activity, would help understand their role in GATORopathies.

Given the variable phenotypes and the relatively common finding of *VUSs* in NRE, it may be arguable whether the *NPRL3* variant can be causative in itself. In this regard, exome sequencing or, even better, whole‐genome sequencing should find a broader application in this condition, to exclude other gene candidates, should they be contributing to *NPRL3* pathogenic mechanism or rather be alternative more plausible causes. The only genome sequencing reported in the *NRE* cohort allowed us to identify a causative deletion in *NPRL3*. In our patient, we did not detect potentially damaging variants in other disease‐related genes, supporting the relevance of the *NPRL3* variant in determining the neurological phenotype. Considering the complexity of *NPRL3*‐related disorder, we cannot exclude that the genetic background of the patient plays a relevant role in influencing penetrance and expressivity.

*NPRL3*‐related epilepsy is slightly more common among females and a similar gender disparity was reported in the prevalence of *DEPDC5* and *NPRL2*‐related epilepsy.^18^ Intellectual disability, developmental delay, and/or language delay occur in a minority of patients (8/74–10.8%). Baldassari in 2019^18^ reported 83 patients with “language or speech delay” or “intellectual disability” out of 183 (45%), among which 75 had DEPDC5 mutations (48% of total DEPDC5 variant—155), 4 NPRL2 (40% of NPRL2 variants—10), and 4 *NPRL3* (4/18, 22% of *NPRL3* variants—18). The divergent prevalence values regarding cognitive and neurodevelopmental issues in patients with GATOR1‐variants might be explained by the fact that these data come from studies centered on the characterization of epileptic manifestations, where the reporting of such comorbidities is less relevant. Given the inconstant association of intellectual disability or neurodevelopmental disorders with GATORopathies, their association with GATOR‐related genes remains less straightforward compared with epilepsy. However, as discussed above, no additional variants were reported in NRE cases that could alternatively explain such comorbidities. Nonetheless, neurodevelopmental abnormalities and epilepsy may share common pathophysiological mechanisms and co‐occur in several known conditions (ie, *CHD2* ^37^ and *GRIN* ^38^). More complex is the case of neuropsychiatric comorbidities such as autism spectrum disorder, that has been reported in many patients with GATORopathies, including NRE.^15^, ^18^ Indeed, neuropsychiatric comorbidities do not seem to correlate exclusively to the epileptic burden, being associated either with drug‐responsive focal epilepsies or severe epileptic phenotypes such as infantile spasms and drug‐resistant epilepsy.

The majority of patients harboring *NPRL3* variants present with frontal lobe epilepsy, either alone or in the context of a SHE or FFEVF syndrome. This finding, which was suggested in a previous review based on a limited number of NRE patients,^18^ has emerged from our review as well. The preferential frontal seizure onset might therefore constitute a peculiar feature of NRE, which would differentiate this entity from other GATOR1‐related epilepsy phenotypes.^18^ The report of additional NRE cases will play a crucial role to confirm this aspect.

Sleep‐related hypermotor epilepsy syndrome was diagnosed in 14.9% (11/74) subjects and FFEVF was observed in 39% (29/74) patients, being reported in four multiplex families with frontotemporal epilepsy.^16^, ^17^, ^33^, ^36^ However, the prevalence of the SHE phenotype might be underestimated due to the concomitant high prevalence of focal seizures with frontal lobe onset and the even higher prevalence of epileptiform abnormalities in the frontal regions (32/67–47.7%), according to the reported ratio (3/7–43%).^18^ Also, the latest definition of SHE encompasses extrafrontal‐onset seizures, making the underestimation of such a diagnosis even more likely. Similarly, the diagnosis of FFEVF usually requires the study of an entire family, whereas many of the reported *NPRL3* variants have been identified in single individuals with poorly characterized family history and pedigrees. Overall, these limitations suggest that the prevalence of FFEVF might be higher than so far reported.

EEG findings cannot evoke a priori an *NPRL3* variant, although specific features, such as activation during sleep or focal fast rhythms in patients with electroclinical picture indicative of FFEVF or SHE may support the interpretation of a genetic result showing a *VUS* in *NPRL3*.

Malformations of cortical developments are common in *NPRL3* patients, especially FCD 2a, and their prevalence is comparable to DEPDC5‐related epilepsy.^18^ It remains unanswered how heterozygous germline mutations (as those listed in Table 2) lead to focal brain lesions. It has been hypothesized that somatic second‐hit mutations limited to certain clusters of neurons, a specific gyrus, or even an entire hemisphere, may occur. After the first observations of pathogenic somatic variants of *MTOR* in FCD^39^, ^40^ the possibly pathogenic involvement of GATOR1 genes in MCDs has ended up under the spotlight. The hyperactivation of mTOR pathway might be the common pathogenetic background of a spectrum of MCDs ranging from the bottom‐of‐sulcus dysplasia^41^ to FCD^42^, ^43^, ^44^, ^45^ and HME.^46^

Among the 15 patients who underwent epilepsy surgery, all had drug‐resistant epilepsy and all but one had a focal lesion detected in MRI. The type of epilepsy was either focal epilepsy or infantile spasms (the latter associated with hemimegalencephaly). Despite the limited number of surgical NRE cases so far, not surprisingly, we could observe a better prognosis in those with a focal lesion identified in MRI. Persistent drug‐resistant seizures after surgery have been reported in three cases: a polymicrogyria resection, a FCD resection, and an MRI‐negative case treated with a DBS implant. It may be supposed that the extension of the primitive lesion and thus residual tissue in the former cases and the MRI negativity in the latter might have impacted the outcome.

Overall, despite the small number of NRE patients treated with epilepsy surgery, a good surgical outcome has been reported, especially in subjects with FCD. Indeed, this specific malformation exhibits favorable characteristics in terms of surgical removals, such as the defined margins. Of note, the frequent localization of FCD in highly eloquent areas within the frontal cortex does not appear to be a significant limitation to the employment of epileptic surgery in these patients. Such a favorable surgery outcome, already described in GATORopathies (DEPDC5‐, NPRL2‐, and *NPRL3*‐related epilepsy),^18^ stands in favor of the presurgical sequencing of GATOR1 genes in subjects with either non‐lesional or FCD‐like imaging patterns. Indeed, this can help for prognostic and diagnostic purposes, including the search for an occult MCD.^47^

Monotherapy is the most common treatment regimen, leading to good seizure control and seizure freedom in up to 18% (11/60) of patients. So far, sirolimus has been employed in drug‐resistant subjects as an add‐on therapy, leading to either transient or no improvement in seizure frequency. However, due to the limited number of patients receiving this drug, further studies are necessary to conclude its efficacy in NRE. Of note, subjects diagnosed with FCD2a may have a better chance to become seizure‐free after epilepsy surgery in comparison to those with a reportedly normal brain MRI and receiving one or more ASMs. This observation further supports the need for a neuroimaging focused on the identification of structural abnormalities in *NPRL3* patients, toward the early identification of good candidates for epilepsy surgery. However, only a few NRE patients undergoing surgery have been reported so far, making it difficult to draw any conclusion on the role of epilepsy surgery in this condition. Also, it is still unclear whether genetic mosaicism, the mechanism likely underlying the heterogenous phenotype of NRE (with and without MCD), may subtend a good surgical outcome.^48^

Among the diverse clinical phenotypes associated with loss‐of‐function variants in GATOR1 genes, *NPRL3*‐related SUDEP (sudden unexpected death in epilepsy) is of particular interest for pathophysiological reasons and prognostic implications.^16^, ^18^, ^49^ Two subjects in our family died. Collectively, inactivating mutations in GATOR1 complex genes explain around 11% of focal epilepsies, whereas no pathogenic mutations were found in GATOR2 complex genes.^16^ GATOR1‐related focal epilepsies differ clinically from focal epilepsies due to mutations in ion channel genes by their association with FCD and seizures emerging from variable foci and nonetheless might confer an increased risk of sudden unexplained death in epilepsy (SUDEP). Nevertheless, the literature review does not allow us to infer conclusions, due to the limited number of reported patients and the rarity of this event. Further reports of large case series will play a relevant role in the elucidation of the frequency and epileptic phenotype in patients with *NPRL3*‐related SUDEP.

## 5 CLINICAL RELEVANCE AND FUTURE DIRECTIONS

*NPRL3*‐related epilepsy is more and more emerging as a potentially distinctive entity within the spectrum of GATOR1‐related epileptic disorders. We reported a novel multiplex family with NRE caused by a novel frameshift variant in *NPRL3* inherited from an unaffected parent, which is consistent with the incomplete penetrance often observed in GATORopathies. Reviewing the current literature, including all reported subjects with NRE, we also provided a detailed phenotypic characterization of the epileptic manifestations observed in this disorder. *NPRL3*‐related seizures respond to ASMs in around one‐third of cases, while evidence in favor of the efficacy of mTOR inhibitors remains elusive. Brain MRI is normal in most of these patients. However, GATOR1 variants can be associated with MCDs and this observation justifies the need for a detailed neuroimaging study in NRE patients. Preliminary data suggest that epilepsy surgery might be a therapeutic approach in NRE patients with MCDs. Long‐term follow‐up data of patients with GATOR‐1 variants and MCDs treated with surgery will define the role of surgery in this condition. The further elucidation of the NRE genotype–phenotype spectrum and understanding of the underlying pathogenetic mechanisms will play a pivotal role in the development of patient‐tailored therapeutical approaches.

## AUTHOR CONTRIBUTIONS

Alice Dainelli contributed to the writing—original draft, review, and editing. Michele Iacomino, Mariasavina Severino, and Valeria Capra contributed to the investigation—data analysis. Sara Rossato and Samuela Bugin contributed to the investigation—clinical assessment and writing—review. Monica Traverso, Stefano Gustincich, and Marco Di Duca contributed to the investigation—genetic testing. Federico Zara contributed to the writing—review and editing. Marcello Scala contributed to the visualization, supervision, writing—original draft, review and editing. Pasquale Striano contributed to the supervision, writing—review and editing.

## FUNDING INFORMATION

This study was supported by “Ricerca Corrente 2023” at IGG and NEXTGENERATIONEU (NGEU) and funded by the Ministry of University and Research (MUR), National Recovery and Resilience Plan (NRRP), project MNESYS (PE0000006)—A Multiscale integrated approach to the study of the nervous system in health and disease (DN. 1553 11.10.2022).

## CONFLICT OF INTEREST STATEMENT

The authors declare that there is no conflict of interest. We confirm that we have read the Journal's position on issues involved in ethical publication and affirm that this report is consistent with those guidelines.
